# Effects of aging on the association between cerebrovascular responses to visual stimulation, hypercapnia and arterial stiffness

**DOI:** 10.3389/fphys.2014.00049

**Published:** 2014-02-19

**Authors:** Daniela Flück, Andrew E. Beaudin, Craig D. Steinback, Gopukumar Kumarpillai, Nandavar Shobha, Cheryl R. McCreary, Stefano Peca, Eric E. Smith, Marc J. Poulin

**Affiliations:** ^1^Department of Biology, Institute of Human Movement Sciences and Sport, ETH ZurichZurich, Switzerland; ^2^Department of Physiology and Pharmacology, Faculty of Medicine, University of CalgaryCalgary, AB, Canada; ^3^Department of Clinical Neurosciences, Faculty of Medicine, University of CalgaryCalgary, AB, Canada; ^4^Hotchkiss Brain Institute, Faculty of Medicine, University of CalgaryCalgary, AB, Canada; ^5^Department of Radiology, Faculty of Medicine, University of CalgaryCalgary, AB, Canada; ^6^Seaman Family MR Research Centre, Foothills Medical Centre, Alberta Health ServicesCalgary, AB, Canada; ^7^Faculty of Kinesiology, University of CalgaryCalgary, AB, Canada; ^8^The Libin Cardiovascular Institute of Alberta, Faculty of Medicine, University of CalgaryCalgary, AB, Canada

**Keywords:** aging, cerebral blood flow, physiology, transcranial doppler, arterial stiffness

## Abstract

Aging is associated with decreased vascular compliance and diminished neurovascular- and hypercapnia-evoked cerebral blood flow (CBF) responses. However, the interplay between arterial stiffness and reduced CBF responses is poorly understood. It was hypothesized that increased cerebral arterial stiffness is associated with reduced evoked responses to both, a flashing checkerboard visual stimulation (i.e., neurovascular coupling), and hypercapnia. To test this hypothesis, 20 older (64 ± 8 year; mean ± *SD*) and 10 young (30 ± 5 year) subjects underwent a visual stimulation (VS) and a hypercapnic test. Blood velocity through the posterior (PCA) and middle cerebral (MCA) arteries was measured concurrently using transcranial Doppler ultrasound (TCD). Cerebral and systemic vascular stiffness were calculated from the cerebral blood velocity and systemic blood pressure waveforms, respectively. Cerebrovascular (MCA: young = 76 ± 15%, older = 98 ± 19%, *p* = 0.004; PCA: young = 80 ± 16%, older = 106 ± 17%, *p* < 0.001) and systemic (young = 59 ± 9% and older = 80 ± 9%, *p* < 0.001) augmentation indices (AI) were higher in the older group. CBF responses to VS (PCA: *p* < 0.026) and hypercapnia (PCA: *p* = 0.018; MCA: *p* = 0.042) were lower in the older group. A curvilinear model fitted to cerebral AI and age showed AI increases until ~60 years of age, after which the increase levels off (PCA: *R*^2^ = 0.45, *p* < 0.001; MCA: *R*^2^ = 0.31, *p* < 0.001). Finally, MCA, but not PCA, hypercapnic reactivity was inversely related to cerebral AI (MCA: *R*^2^ = 0.28, *p* = 0.002; PCA: *R*^2^ = 0.10, *p* = 0.104). A similar inverse relationship was not observed with the PCA blood flow response to VS (*R*^2^ = 0.06, *p* = 0.174). In conclusion, older subjects had reduced neurovascular- and hypercapnia-mediated CBF responses. Furthermore, lower hypercapnia-mediated blood flow responses through the MCA were associated with increased vascular stiffness. These findings suggest the reduced hypercapnia-evoked CBF responses through the MCA, in older individuals may be secondary to vascular stiffening.

## Introduction

Age manifests in systemic decreases in vascular compliance leading to an increased risk of stroke, cerebral white matter lesions, and cognitive decline (Mitchell et al., 2011; Laurent et al., 2012; Poels et al., 2012; Xu et al., 2012). In the brain, decreased resting cerebral blood flow (CBF) and cerebrovascular reactivity to neuronal activation and alterations in arterial blood gases are also associated with an elevated risk of cerebrovascular disease (Jennings et al., 2013) and occur with healthy aging (Nishiyama et al., 1997; Fisher et al., 2013). Thus, the concurrent increase in cerebrovascular stiffness with aging may impact cerebrovascular responses (Fonck et al., 2009; Zhu et al., 2011) and thus contribute to the decreased CBF responses to neuronal stimulation and hypercapnia that occur with aging.

CBF is elevated in response to increased neural activity (i.e., neurovascular coupling) and hypercapnia. Neural activation leads to local increases in CBF via functional hyperemia, whereas hypercapnia produces a global increase in CBF, although there is heterogeneity between brain regions (Noth et al., 2008). With aging, resting CBF, neuronal-mediated increases in CBF, and CBF responses to hypercapnia have all been reported to decrease (Panczel et al., 1999; Niehaus et al., 2001; Fisher et al., 2013; Jennings et al., 2013), but this is not a consistent finding with other studies reporting no change in neurovascular coupling (Rosengarten et al., 2003) or reactivity to hypercapnia (Schwertfeger et al., 2006; Galvin et al., 2010), and one study even reporting greater hypercapnia reactivity with healthy aging (Zhu et al., 2013). As such, the mechanisms regulating changes in resting CBF and CBF reactivity in response to neuronal activation and hypercapnia with aging are incompletely understood. Contributing mechanisms likely include brain atrophy, altered neuronal activity, and decreased cerebral metabolism (Leenders et al., 1990; Fisher et al., 2013), although endothelial and hemodynamic alterations may also contribute (Secher et al., 2008; Zhu et al., 2011; Fisher et al., 2013).

To assess neural-evoked increases in CBF, the approach of monitoring blood flow through the posterior cerebral artery (PCA) in response to a visual stimulus has been used extensively (Aaslid, 1987; Sturzenegger et al., 1996; Spelsberg et al., 1998; Panczel et al., 1999; Niehaus et al., 2001; Zaletel et al., 2004; Lisak et al., 2005; Smith et al., 2008; Rey et al., 2010), as the PCA supplies the majority of blood to the visual cortex (Edvinsson and Krause, 2002). Moreover, concurrent monitoring of middle cerebral artery (MCA) blood flow can be used as a negative control to confirm the locality of the visually-induced increase in blood flow through the PCA during such challenges since the MCA blood flow response to the same challenges is minimal (Aaslid, 1987; Smith et al., 2008).

The cerebral circulation is exquisitely sensitive to changes in the arterial partial pressure of carbon dioxide (Pa_CO_2__) (Berne et al., 1981; Poulin and Robbins, 1996), increasing with hypercapnia and decreasing with hypocapnia. In humans, blood velocity through both the PCA and MCA increase ~3–5% per mmHg increase in Pa_CO_2__ above resting values (Tominaga et al., 1976; Ide et al., 2003), and thus reflect the global influence of hypercapnia on CBF.

Transcranial Doppler ultrasound (TCD) is a useful non-invasive technique to assess both neural- and hypercapnia-mediated changes in CBF as well as cerebral hemodynamics on a beat-to-beat basis because of its high temporal resolution. Changes in CBF are typically monitored via changes in the peak velocity envelope averaged across a heartbeat while cerebral hemodynamics may be assessed by examining specific parameters of the peak blood velocity waveform (Robertson et al., 2008). In turn, these variables can provide measures of CBF reactivity to specific stimuli and cerebrovascular health, respectively.

Analogs to examining the pulse pressure waveform (Wilkinson et al., 1998), analysis of the TCD peak blood velocity waveform can be exploited to obtain a measure of arterial stiffening via calculation of a cerebral augmentation index (AI) (Kurji et al., 2006; Robertson et al., 2008). The AI is based on wave reflections throughout the vascular bed caused by vessel branching, changes in vessel wall diameter and/or material properties (Mitchell et al., 2011). The arrival of the reflected wave depends on the site of reflection as well as on the stiffness of the respective vessel being monitored. The stiffer the vessel, the higher the velocity of the forward and backward travelling waves, which leads to an earlier arrival of the reflected waves. The earlier arrival of the reflected wave is superimposed on the forward travelling wave and consequently higher velocity at the so-called reflective point of the peak blood velocity waveform (Laurent et al., 2006). Thus, an increase in AI is observed as vessels downstream of the monitored artery become less distensible with increasing age (Benetos et al., 1993).

Although mechanisms underlying neural- and hypercapnia-mediated vasodilation are unique to each stimulus, as both may be reduced with age, there is likely a common mechanism contributing to the decrease observed in each with increasing age. We speculate this common mechanism is age-related vascular mechanical dysfunction (i.e., reduced distensibility). As such, it was hypothesized that resting CBF and neural- and hypercapnia-evoked increases in CBF would be attenuated with age, and attenuated neural- and hypercapnia-evoked CBF increases would be associated with elevated arterial stiffness (i.e., AI) within the PCA and MCA, respectively. To test this hypothesis, the relationship between CBF responses to a visual stimulus and a hypercapnic challenge with AI were examined and compared between young and older humans.

## Methods

### Subjects

Thirty subjects, 20 older (12 men; 8 women) and 10 younger (4 men, 6 females) participated in this study (Table 1). Exclusion criteria included age <55 y for the older group and age <18 or >55 y for the younger group, recent (<60 days) change in blood pressure medication, uncontrolled hypertension, history of stroke, neurological disease, or dementia [defined according to the Diagnostic and Statistical Manual of Mental Disorders (American Psychiatric Association, 2000)]. A neurologist (Eric E. Smith) obtained clinical and medication histories, performed a neurological examination, and administered the Mini-Mental Status Examination (MMSE) cognitive test to all older volunteers (Folstein et al., 1975). Volunteers scoring <23 on the MMSE were excluded. Prior to experimental testing, subjects provided written and oral informed consent. The study was approved by the University of Calgary Conjoint Health Research Ethics Board.

**Table 1:** **Demographics of the study cohort and resting end-tidal partial pressures of O_2_ and CO_2_, and cerebral hemodynamic indices in the posterior and middle cerebral arteries**.

	**Young**	**Older**	***p*-values**
Age, years	30 ± 5	64 ± 8	***p* < 0.001**
Height, cm	171 ± 9	169 ± 0	*p* = 0.552
Weight, kg	67 ± 13	77 ± 15	*p* = 0.084
SBP, mmHg	112 ± 9	120 ± 18	*p* = 0.188
DBP, mmHg	66 ± 6	68 ± 8	*p* = 0.571
MAP, mmHg	82 ± 6	85 ± 10	*p* = 0.383
Systemic AI, %	59.0 ± 9.4	80.3 ± 9.0	***p* < 0.001**
Pet_O_2__, Torr	85.3 ± 3.7	88.6 ± 6.4	*p* = 0.149
Pet_CO_2__, Torr	35.2 ± 3.3	33.2 ± 3.3	*p* = 0.133
**POSTERIOR CEREBRAL ARTERY**			
Sample size (n)	10	18	
*V*_dia_, cm/s	22.6 ± 5.0	21.3 ± 5.0	*p* = 0.514
*V*_sys_, cm/s	53.3 ± 12.0	49.7 ± 8.4	*p* = 0.354
*V*_mean_, cm/s	34.4 ± 7.5	33.7 ± 7.2	*p* = 0.829
*V*_refl_, cm/s	46.3 ± 9.4	51.6 ± 11.6	*p* = 0.224
Cerebral AI, %	80.5 ± 15.8	106.8 ± 16.8	***p* < 0.001**
CVC, cm/s/mmHg	0.43 ± 0.11	0.41 ± 0.12	*p* = 0.694
**MIDDLE CEREBRAL ARTERY**			
Sample size (n)	10	19	
*V*_dia_, cm/s	40.0 ± 5.2	28.4 ± 7.4	***p* < 0.001**
*V*_sys_, cm/s	94.2 ± 18.2	68.1 ± 11.8	***p* < 0.001**
*V*_mean_, cm/s	60.0 ± 8.8	44.5 ± 9.3	***p* < 0.001**
*V*_refl_, cm/s	79.8 ± 13.7	66.8 ± 14.0	***p* = 0.024**
Cerebral AI, %	76.1 ± 15.5	98.0 ± 19.0	***p* = 0.004**
CVC, cm/s/mmHg	0.74 ± 0.15	0.54 ± 0.13	***p* < 0.001**

Values are means ± SD.

*SBP, systolic blood pressure; DBP, diastolic blood pressure; MAP, mean arterial blood pressure; systemic AI, systemic augmentation index; P*et*_CO_2__, end-tidal partial pressure of CO_2_; P*et*_O_2__, end-tidal partial pressure of O_2_; V_dia_, velocity at diastole; V_sys_, velocity at systole; V_mean_, average velocity over cardiac cycle; V_refl_, velocity at the reflected shoulder of peak systole; cerebral AI, cerebral augmentation index; CVC, cerebrovascular conductance; p-values: older compared to young. The bold p-values are to highlight the significant differences that were observed.*

### Instrumentation

Bilateral TCD was used to measure PCA and MCA blood velocity simultaneously with subjects in a semi-supine position. The P2 segment of the PCA was monitored on the ipsilateral side of the dominant hand while the M2 segment of the MCA was monitored on the contralateral side. TCD probes were held in position by snug-fitting headgear (marc600, Spencer Technologies, Seattle, WA). Heart rate was measured using a 3-lead ECG (Micromon 7142B, Kontron Medical, Milton Keynes, UK); continuous arterial blood pressure was recorded non-invasively via finger photoplethysmography (Portapres, TPD Biomedical Instrumentation, Amsterdam, Netherlands) and intermittently from the brachial artery via an automated cuff (DINAMAP compact5, Critikon, New Jersey, USA); and arterial oxyhemoglobin saturation was collected via finger pulse oximetry (Datex-Ohmeda 3900, Helsinki, Finland). End-tidal partial pressures of oxygen (Pet_O_2__) and carbon dioxide (Pet_CO_2__) were monitored via a nasal cannula while subjects performed a visual stimulation test. Next, a euoxic hypercapnic test was administered using the technique of dynamic end-tidal forcing (DEF) with the subject breathing through a mouthpiece with their nose occluded (Ide et al., 2003). The DEF system uses a negative feedback loop to control Pet_CO_2__ and Pet_O_2__ at desired levels by adjusting the inspired fraction of CO_2_ (F_I_CO_2_) and O_2_ (F_I_O_2_) on a breath-by-breath basis using custom designed, dedicated software (BreatheM, v2.38, University Laboratory of Physiology, Oxford, UK) (Vantanajal et al., 2007; Beaudin et al., 2011). The DEF system controls Pet_CO_2__ and Pet_O_2__ at desired levels independent of ventilatory frequency and depth. Respired gases were sampled at 20 mL/min via a fine capillary inserted into the nasal cannula during the visual stimulation test or the mouthpiece during the euoxic hypercapnic test for continuous analysis of F_I_CO_2_ and F_I_O_2_ by mass spectrometry (AMIS 2000, Innovision, Odense, Denmark).

### Visual stimulation

To assess the CBF response to visual stimulation, the subject sat ~50 cm from a 38.1 cm (i.e., 15 in) computer screen with their eyes in line with its center when looking straight ahead (Smith et al., 2008). Following instrumentation, the test started with a 2-min baseline consisting of looking at a black cross (height and width ~1.5 cm) centered on a dark gray background. Baseline was followed by either 10 cycles of 40 s blocks (*n* = 9 older subjects) or 5 cycles of 80 s blocks (*n* = 11 older subjects and all young subjects) involving the flashing of an alternating black and white checkerboard stimulus for 20 or 40 s (ON), respectively. The ON stage was followed by 20 or 40 s of rest (OFF) where the screens display was the same as baseline conditions. The subject was instructed to always focus their eyes on the computer screen and their thoughts on the present task, and was monitored continuously to verify they were attending to the stimulus. All tests were performed in a darkened room.

This visual stimulation paradigm was chosen to minimize participant burden as it was also used with elderly stroke patients (data not reported), some of whom had mild dementia (Peca et al., 2013) and difficulty following relatively simple tasks. Therefore, using the current visual stimulation paradigm simplified the task by removing the necessity of continuously instructing subjects when to open and close their eyes.

### Euoxic hypercapnic test

The euoxic hypercapnic test began with 10 min of air breathing to determine mean resting Pet_CO_2__ and Pet_O_2__ values. These mean resting end-tidal values were used to create an individualized hypercapnic protocol (Ide et al., 2003). The euoxic hypercapnic test consisted of three 120 s steps: Baseline (Pet_CO_2__ = +1.5 Torr above resting values), Hypercapnia (Pet_CO_2__ = +6.5 Torr above resting values), and Recovery (Pet_CO_2__ = +1.5 Torr above resting values). Throughout the entire hypercapnic test, Pet_O_2__ was maintained at 88 Torr (mean euoxic Pet_O_2__ for the altitude (1103 m above sea level) at which the laboratory is located). Maintaining Pet_CO_2__ at +1.5 Torr above air breathing resting values during the Baseline and Recovery facilitates Pet_CO_2__ control (Ide et al., 2003) and reduces breath-to-breath variability in CBF velocity (Harris et al., 2006).

### Analysis

The peak velocity associated with the maximal Doppler frequency shift was averaged over each heart beat (*V*_mean_; cm/s) and utilized as an index of CBF through the PCA and MCA.

**Figure 1:**
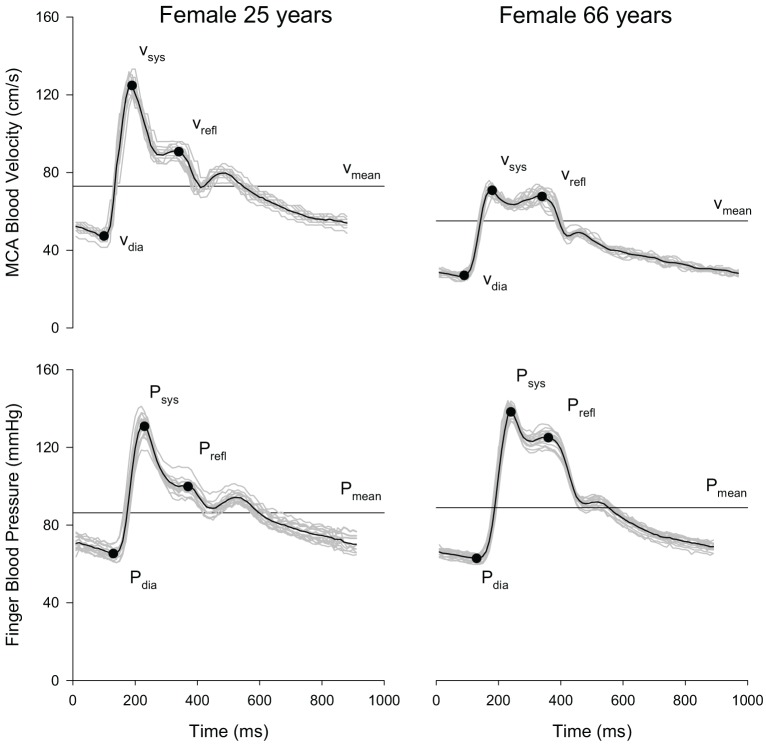
**Representative MCA blood velocity and finger blood pressure waveforms of a young (age = 25 years) and older (age = 66 years) volunteer at rest.** Each plot shows the overlay of individual cardiac cycles (gray lines) over 15 s, the composite (i.e., mean waveform; black line), and the location of the waveform parameters.

Velocity waveform analysis was performed using a custom written Matlab program (Robertson et al., 2008). Output parameters included the diastolic minimum velocity (*V*_dia_), velocity at systole (*V*_sys_), velocity at the reflective shoulder (*V*_refl_), and mean peak velocity (*V*_mean_) (Figure 1). Next, the cerebral augmentation index (AI) was calculated (Kurji et al., 2006; Robertson et al., 2008):

(1) $$\text{Cerebral AI}=\frac{V_{\text{refl}}-V_{\text{dia}}}{V_{\text{sys}}-V_{\text{dia}}}\times 100$$

Finally, utilizing mean arterial pressure (MAP), beat-to-beat PCA and MCA cerebrovascular conductance (CVC) was calculated as:

(2) $$\text{CVC}=\frac{V_{\text{mean}}}{\text{MAP}}$$

Visual waveform analysis was performed using the final 60 s of baseline and the final 30 s of a minimum of three ON phases (15 s for 40 s blocks). For the hypercapnic test, the final 60 s of the baseline and hypercapnic stage were analyzed to determine hypercapnic responses. Absolute blood flow changes in response to visual stimulation and hypercapnia were calculated by subtracting the baseline values from the ON phase and hypercapnia values, respectively. In addition, the relative (i.e., percent) change in blood flow from the preceding baseline in response to the visual stimulation and hypercapnia were calculated. Subsequently, PCA and MCA reactivity to hypercapnia was quantified in absolute (cm/s/Torr) and relative (%/Torr) units by dividing the respective increases in PCA and MCA blood velocity by the change in PET_CO_2__ from baseline into hypercapnia.

Systemic pressure waveform parameters were analyzed using the same automated algorithms implemented in Matlab used to analyze the velocity waveforms. Systemic AI was calculated analogically to the cerebral AI using systolic blood pressure (*P*_sys_), diastolic blood pressure (*P*_dia_) and blood pressure at the reflective shoulder (*P*_refl_).

(3) $$\text{Systemic AI}=\frac{P_{\text{refl}}-P_{\text{dia}}}{P_{\text{sys}}-P_{\text{dia}}}\times 100$$

### Statistical analysis

All measured parameters had a normal distribution as assessed by the Kolmogorov-Smirnov Test. Differences in subject demographics and resting cerebrovascular hemodynamic parameters were compared using independent Student *T*-Tests. Next, within- and between-subject differences in responses to the visual stimulation and euoxic hypercapnia tests were assessed using 2-by-2 mixed factor repeated measures analyses of variance (RM ANOVA). The within-subject factor for the visual stimulation test was Visual Stimulation Stages (OFF and ON) and the within-subject factor for the hypercapnic test was Hypercapnic Test Stages (Baseline and Hypercapnia). The between-subject factor was Age Groups (Young and Older). If there was a significant main effect of Visual Stimulation Stages or Hypercapnic Stages, *post-hoc* within-subject differences in cerebral blood velocity and waveform parameters between the OFF and ON or the Baseline and Hypercapnic stages (irrespective of Age Groups) were compared using paired Student *T*-Tests. If the interaction between the Visual Stimulation Stages or Hypercapnic Test Stages and Age Groups was significant [or showed a trend to be significant (i.e., 0.05 ≤ *p* ≤ 0.100)], the magnitude of the response (i.e., absolute and relative delta values) for each age group were compared using independent 1-tailed Student *T*-Tests. All *post-hoc* analyses incorporated a Bonferroni correction for multiple comparisons.

Based upon previously published reports showing a curvilinear relationship between systemic AI and age (Kelly et al., 1989; Mitchell et al., 2004; McEniery et al., 2005), a similar relationship was plotted between cerebral AI and age. Alpha was set *a priori* at *p* ≤ 0.05. Finally, the Pearson product-moment correlation was used to examine relationships between cerebral and systemic AI, changes in PCA blood velocity in response to the visual stimulation and cerebral AI, and hypercapnic reactivity for the PCA and MCA and cerebral AI. Statistical analyses were performed using SAS Enterprise Guide (4.3, SAS Institute Inc., Cary, NC, USA).

## Results

All study subjects (*n* = 30) completed the study. The TCD signal was of insufficient quality for analysis of PCA blood velocity in two older volunteers and of MCA blood velocity in one older volunteer. Subject demographics, resting blood pressure, resting Pet_O_2__ and Pet_CO_2__, and cerebral blood velocity parameters are presented in Table 1. Briefly, the young and older subjects were of similar height (*p* = 0.552) and weight (*p* = 0.084) and had similar systolic (SBP), diastolic (DBP) and mean arterial (MAP) blood pressures (*p* ≥ 0.188). Additionally, resting, air breathing Pet_O_2__ and Pet_CO_2__ were similar between the two age groups (*p* ≥ 0.133). In contrast, the older group had a significantly higher systemic AI (*p* < 0.001).

### Resting cerebrovascular hemodynamic parameters

At rest, while the subjects were breathing room air, PCA diastolic (*V*_dia_), systolic (*V*_sys_), mean (*V*_mean_), and reflected (*V*_refl_) blood velocities, as well as CVC, were similar between the young and older subjects (*p* ≥ 0.224). Oppositely, PCA cerebral AI was lower in the young group (*p* < 0.001). For the MCA, all blood velocity waveform parameters (i.e., *V*_dia_, *V*_sys_, *V*_mean_, and *V*_refl_) and CVC were higher (*p* ≤ 0.024), and cerebral AI was lower in the young group (*p* = 0.004).

### Responses to visual stimulation

There was no effect of the Visual Stimulation Stages and the interaction between the Visual Stimulation Stages and Age Groups main effects were not significant for Pet_O_2__ (*p* ≥ 0.347), Pet_CO_2__ (*p* ≥ 0.518), or MAP (*p* ≥ 0.107). Thus, there was no change in Pet_O_2__, Pet_CO_2__, and MAP in response to the visual stimulation, irrespective of age, and any changes in Pet_O_2__, Pet_CO_2__, and MAP that occurred within each age group were similar (Table 2).

**Table 2:** **Changes in Pet_CO_2__, Pet_O_2__, MAP, and absolute and relative (i.e., percent) changes in cerebral waveform parameters for the posterior and middle cerebral arteries in response to the visual stimulation and CO_2_ test in the young and older groups**.

	**Visual stimulation**			**CO_2_ Test**		
	**Young**	**Older**	***p*-values**	**Young**	**Older**	***p*-values**
ΔPet_CO_2__ (Torr)	0.2 ± 1.0	0.1 ± 1.1	*p* = 0.681	4.5 ± 1.0	4.7 ± 1.6	*p* = 0.728
ΔPet_O_2__ (Torr)	−1.0 ± 2.7	−0.3 ± 3.5	*p* = 0.585	−0.4 ± 2.4	−0.4 ± 2.4	*p* = 0.970
ΔMAP	−2.2 ± 2.8	1.42 ± 6.5	*p* = 0.107	4.3 ± 4.2	3.6 ± 4.9	*p* = 0.712
**POSTERIOR CEREBRAL ARTERY**						
Sample size (n)	10	18		10	18	
Δ*V*_dia_, cm/s	3.6 ± 1.6	2.2 ± 1.6	***p* = 0.016**	6.2 ± 1.3	3.4 ± 2.0	***p* < 0.001**
Δ*V*_dia_, %	16.6 ± 7.7	11.3 ± 8.8	*p* = 0.059	24.0 ± 7.5	15.7 ± 8.6	***p* = 0.009**
Δ*V*_sys_, cm/s	5.3 ± 3.6	2.7 ± 2.5	***p* = 0.014**	7.5 ± 4.6	4.7 ± 3.2	***p* = 0.035**
Δ*V*_sys_, %	10.5 ± 7.1	5.6 ± 4.9	***p* = 0.019**	13.7 ± 7.5	10.1 ± 6.2	*p* = 0.088
Δ*V*_refl_, cm/s	5.7 ± 3.1	3.6 ± 3.2	***p* = 0.047**	9.0 ± 3.3	5.9 ± 3.3	***p* = 0.012**
Δ*V*_refl_, %	12.8 ± 7.0	7.2 ± 6.6	***p* = 0.022**	17.1 ± 7.1	11.9 ± 7.3	***p* = 0.041**
**MIDDLE CEREBRAL ARTERY**						
Sample size (n)	10	19		10	19	
Δ*V*_dia_, cm/s	0.6 ± 1.9	1.3 ± 1.9	*p* = 0.174	10.9 ± 3.2	5.6 ± 2.4	***p* < 0.001**
Δ*V*_dia_, %	1.7 ± 5.2	5.8 ± 9.6	*p* = 0.110	23.6 ± 6.4	18.2 ± 6.8	***p* = 0.024**
Δ*V*_sys_, cm/s	−0.3 ± 4.4	1.4 ± 2.3	*p* = 0.093	11.6 ± 5.2	7.9 ± 5.0	***p* = 0.034**
Δ*V*_sys_, %	−0.2 ± 4.7	2.2 ± 3.4	*p* = 0.061	12.0 ± 4.5	11.7 ± 7.4	*p* = 0.465
Δ*V*_refl_, cm/s	0.9 ± 3.7	1.6 ± 3.5	*p* = 0.299	15.7 ± 3.8	10.3 ± 4.8	***p* = 0.002**
Δ*V*_refl_, %	1.2 ± 4.9	2.7 ± 6.3	*p* = 0.263	17.7 ± 3.9	14.9 ± 7.5	*p* = 0.150

Values are means ± SD.

*P*et*_CO_2__, end-tidal partial pressure of carbon dioxide; P*et*_O_2__, end-tidal partial pressure of oxygen; MAP, mean arterial pressure; V_dia_, velocity at diastole; V_sys_, velocity at systole; V_refl_, velocity at the reflected shoulder of peak systole; cerebral AI, cerebral augmentation index; CVC, cerebrovascular conductance.*

P-values within table are for older vs. young comparisons. The bold p-values are to highlight the significant

differences that were observed.

**Figure 2:**
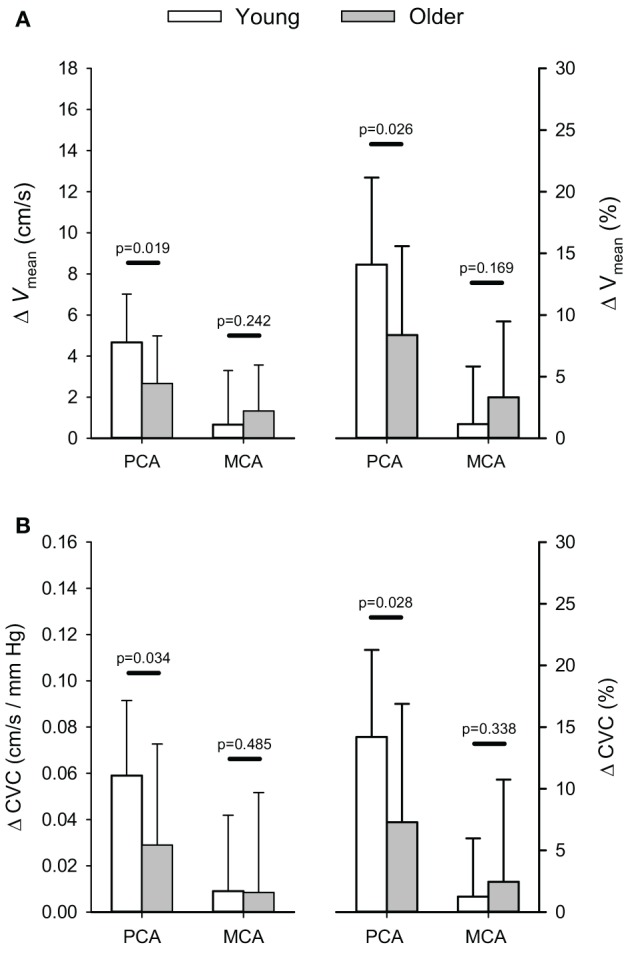
**Absolute (cm/s) and relative (%) changes (Δ) in posterior (PCA) and middle cerebral artery (MCA) blood velocity (A) and cerebrovascular conductance (B) in response to visual stimulation.** *P*-values provided for comparison between young and older volunteers.

In the older group, visually evoked increases in PCA blood velocity were similar between those who performed 10 cycles of 40 s blocks and those who performed 5 cycles of 80 s blocks (*p* = 0.172). Thus, responses were grouped to provide a single group mean. The mean change in absolute, and relative, visually evoked changes in PCA and MCA mean blood velocities (*V*_mean_) and CVC for both the young and older groups are shown in Figure 2. The Visual Stimulation Stages main effect was significant for both PCA (*p* < 0.001), and MCA, *V*_mean_ (*p* = 0.041) with *V*_mean_, being higher during the ON stage for both arteries (*p* ≤ 0.041) regardless of age. The increase in PCA *V*_mean_ was 3.4 ± 2.5 cm/s (10.3 ± 7.6%), which was greater (*p* ≤ 0.001) than the increase in MCA *V*_mean_ of 1.1 ± 2.4 cm/s (2.6 ± 5.7%). Moreover, the interaction between the Visual Stimulation Stages and Age Groups main effects was significant for PCA *V*_mean_ (*p* = 0.037) as a result of a greater increase in absolute (and relative) PCA *V*_mean_ in the young group (*p* ≤ 0.026; Figure 2A). The non-significant interaction between Visual Stimulation Stages and Age Groups for the MCA *V*_mean_ signifies the increase in MCA *V*_mean_ was similar between the two age groups (Figure 2A).

There was a significant effect of the Visual Stimulation Stages on PCA CVC (*p* ≤ 0.001), but not MCA CVC (*p* = 0.381). Regardless of age, PCA CVC was higher during the ON stage of the visual stimulation test (*p* ≤ 0.001). In addition, there was a trend for the interaction between the Visual Stimulation Stages and Age Groups main effects to be significant for PCA CVC (*p* = 0.067) while the interaction was not significant for MCA CVC (*p* = 0.187; Figure 2B). Although the interaction term showed only a trend, *post-hoc* 1-tailed comparisons of the change in PCA CVC (absolute and relative) showed the increase in CVC was greater in the young group (*p* ≤ 0.034; Figure 2B).

Finally, the Visual Stimulation Stages main effect was significant for PCA *V*_dia_, *V*_sys_, and *V*_refl_ (*p* ≤ 0.001) as all velocities were elevated in response to the visual stimuli within the young and older groups. In contrast, the Visual Stimulation Stages main effect was significant for only MCA *V*_dia_ (*p* = 0.015) with it being elevated in response to the visual stimuli (Table 2). Additionally, there was a significant interaction between the Visual Stimulation Stages and Age Groups main effects for PCA *V*_dia_ (*p* = 0.032) and *V*_sys_ (*p* = 0.028) with the young subjects having a greater increase in the two velocities in response to the visual stimuli (Table 2). The interaction was not significant for MCA *V*_dia_, *V*_sys_, and *V*_refl_ (*p* ≥ 0.187).

### Responses to euoxic hypercapnia

Baseline Pet_O_2__ and Pet_CO_2__were 87.8 ± 2.4 Torr and 37.3 ± 3.0 Torr for the young group and 87.8 ± 1.8 Torr and 35.6 ± 3.3 Torr in the older group (*p* ≥ 0.172). Subsequently, Pet_O_2__ and Pet_CO_2__ during the hypercapnic stage were 87.5 ± 2.0 Torr and 41.8 ± 3.2 Torr in the young group and 87.3 ± 1.8 Torr and 40.5 ± 3.0 Torr in the older group (*p* ≥ 0.277). There was no interaction between the Hypercapnic Stages and Age Groups for either Pet_O_2__ or Pet_CO_2__ (*p* ≥ 0.326). Thus, Pet_O_2__ and the increase in Pet_CO_2__, with hypercapnia were similar between young and older subjects (*p* ≥ 0.728; Table 2). For MAP, there was a significant effect the Hypercapnic Stages (*p* < 0.001) as MAP was increased with hypercapnia irrespective of age. The interaction between the Hypercapnic Stages and Age Groups main effects was not significant (*p* = 0.712) as the increase in MAP was similar between young and older subjects (Table 2).

**Figure 3:**
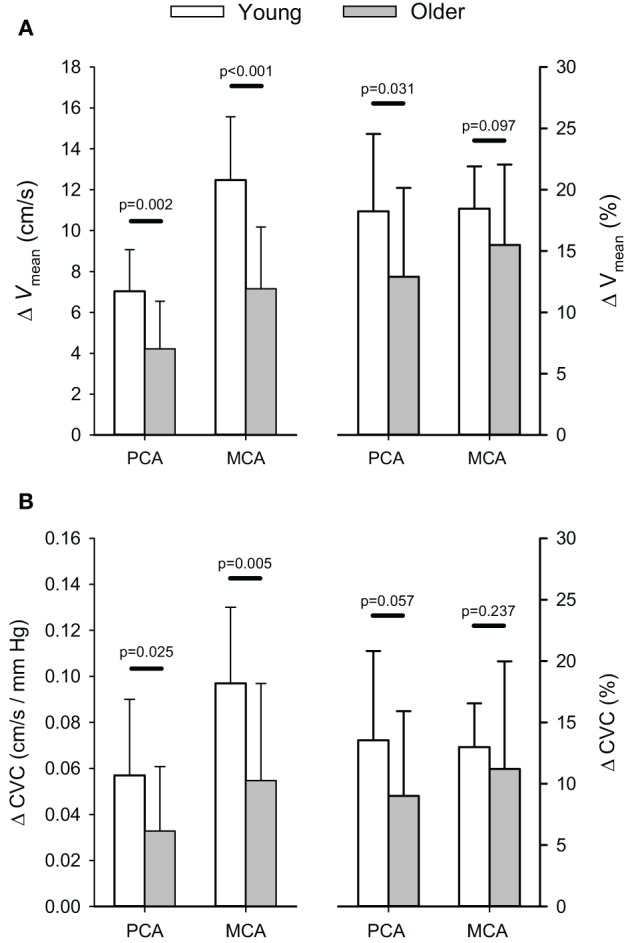
**Absolute (cm/s) and relative (%) changes (Δ) in posterior (PCA) and middle cerebral artery (MCA) blood velocity (A) and cerebrovascular conductance (B) in response to euoxic hypercapnic test.** *P*-values provided for comparison between young and older volunteers.

Absolute and relative increases in PCA and MCA *V*_mean_, and CVC with hypercapnia are shown in Figure 3. There was a significant interaction between the Hypercapnic Stages and Age Groups main effects for both PCA (*p* = 0.004) and MCA (*p* < 0.001) *V*_mean_. In addition, the interaction between the Hypercapnic Stages and Age Groups was significant for CVC in both arteries (PCA: *p* = 0.039; MCA: *p* = 0.007). *Post-hoc* comparisons showed the absolute, and relative, increases in PCA *V*_mean_ were significantly lower in older subjects (*p* ≤ 0.031) while the absolute increase in MCA *V*_mean_ was lower in the older subjects (*p* ≤ 0.001) and the relative increase in MCA *V*_mean_ showed a trend to be lower in the older group (*p* = 0.097). Also, the absolute increase in CVC with hypercapnia was lower in the older group for both the PCA (*p* = 0.025), and MCA (*p* = 0.005), but the relative increase in CVC showed only a trend to be lower in the older group for the PCA (*p* = 0.057) while MCA CVC was similar between the two groups (*p* = 0.237). The interaction between the Hypercapnic Stages and Age Groups main effects was significant for PCA and MCA *V*_dia_ (*p* ≤ 0.001) and *V*_refl_ (*p* ≤ 0.024), but not *V*_sys_ (*p* ≥ 0.068). Table 2 shows the results of *post-hoc* age group comparisons of the change in PCA and MCA *V*_dia_, *V*_sys_, and *V*_refl_ with hypercapnia.

**Figure 4:**
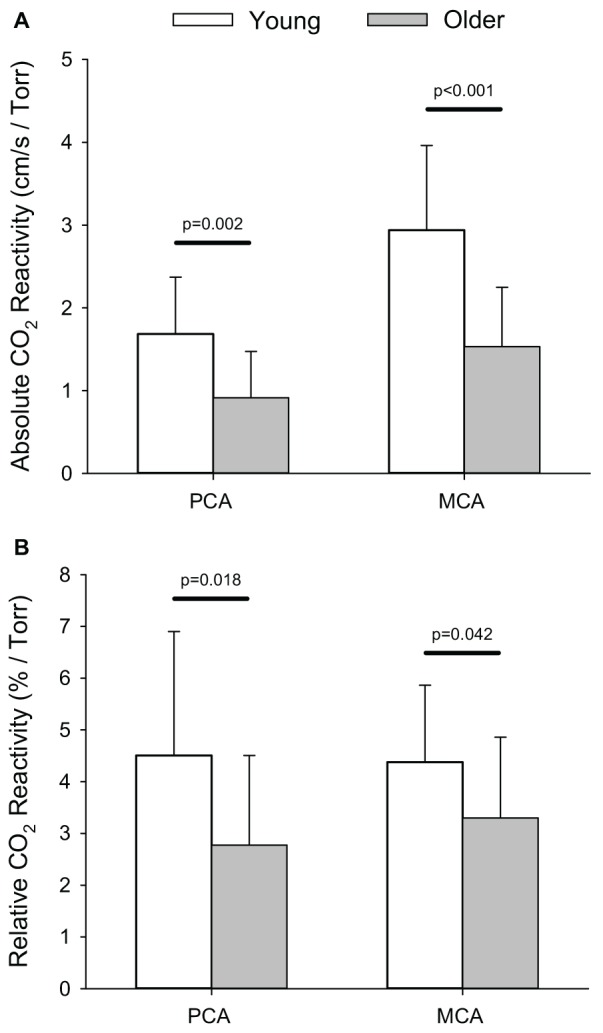
**Absolute (i.e., cm/s/Torr; A) and relative (i.e., %/Torr, B) reactivity for posterior (PCA) and middle cerebral artery (MCA) blood velocity in response to the hypercapnic test.** *P*-values provided for comparison between young and older volunteers.

The smaller absolute, and relative, increases in PCA *V*_mean_ within the older group translated into lower absolute (i.e., cm/s/Torr) and relative (i.e., %/Torr) reactivities to hypercapnia (*p* ≤ 0.018) compared to the young group (Figure 4). Similarly, absolute, and relative, MCA hypercapnia reactivity was lower in the older group (*p* ≤ 0.042). Relative PCA and MCA hypercapnic reactivity values for the older group have been previously reported in comparison to a patient population (Peca et al., 2013).

### Visually-evoked and hypercapnic CBF responses: relation to arterial stiffness

**Figure 5:**
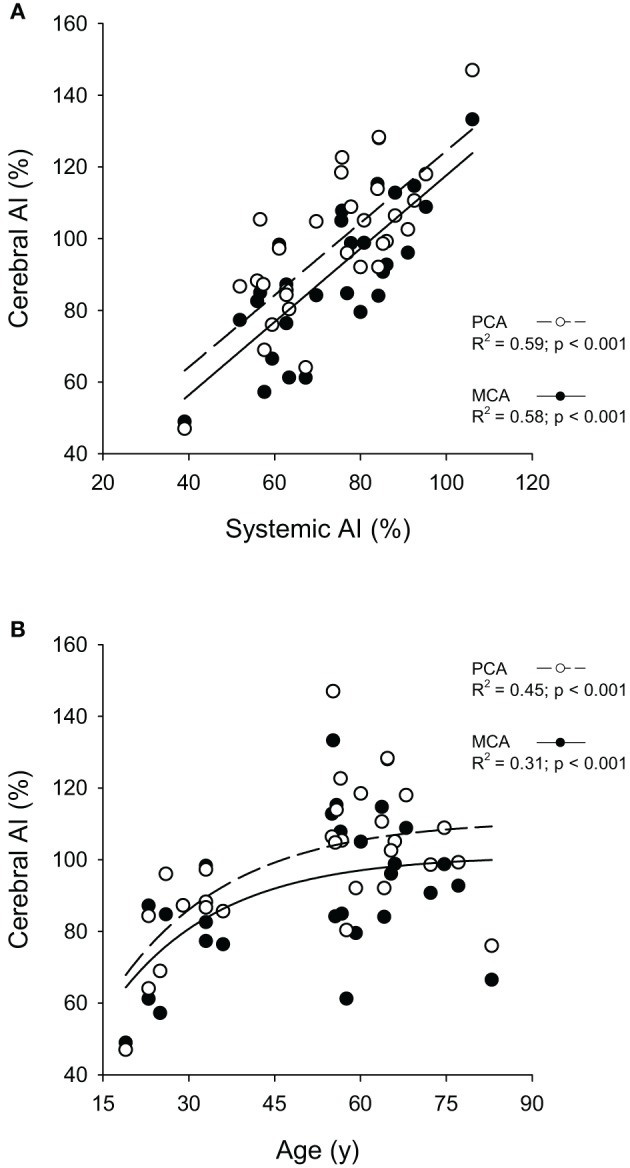
**Relationships between the cerebral augmentation index (Cerebral AI) and Systemic AI in the middle (MCA) and posterior cerebral arteries (PCA) (A) and Cerebral AI and Age in MCA and PCA (B)**.

**Figure 6:**
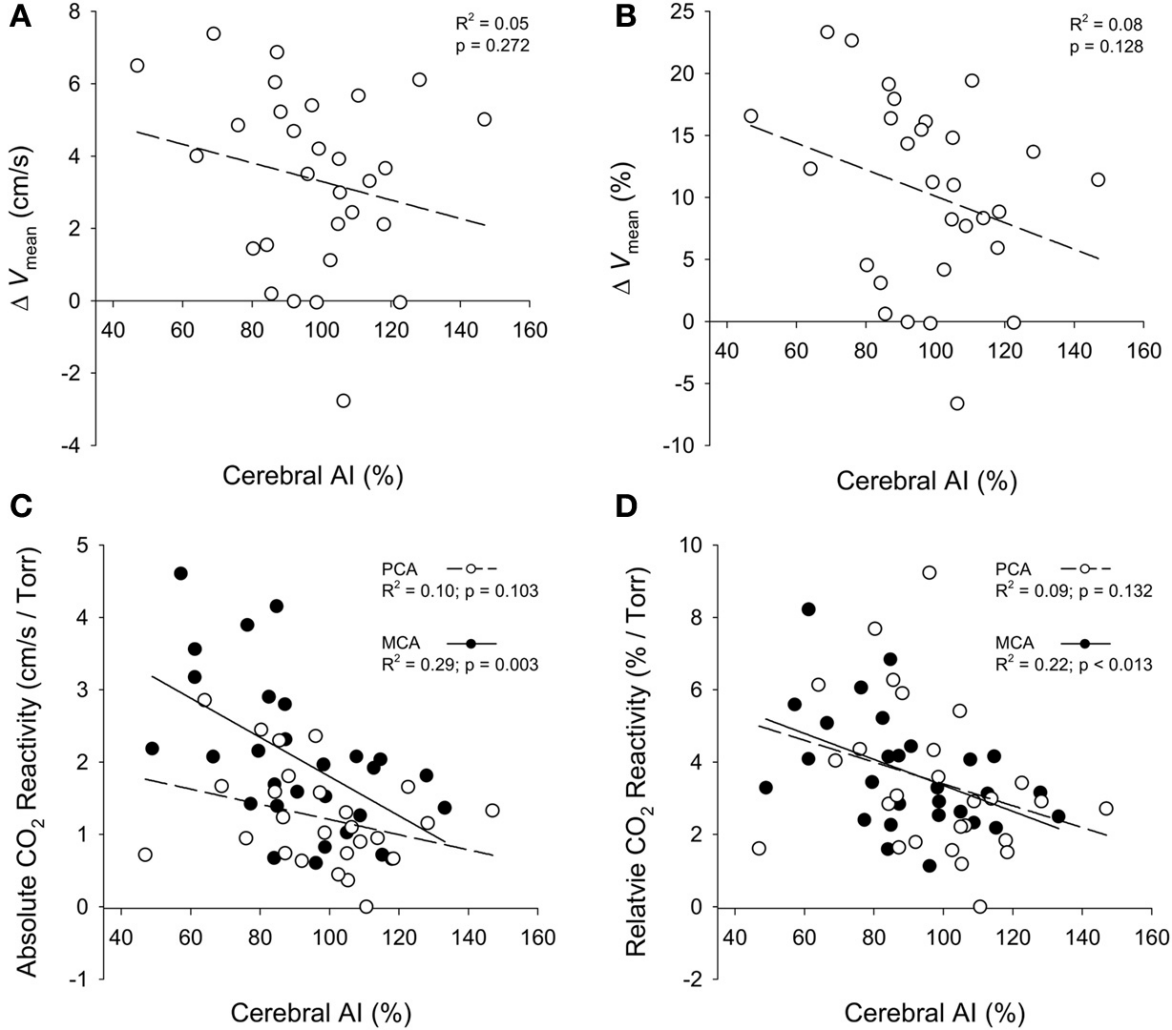
**Relationships between the absolute (cm/s) and relative (%) changes in PCA mean blood velocity (Δ*V*_mean_) in response to the visual stimulation and cerebral AI (A,B); and the relationships between the absolute (cm/s/Torr) and relative (%/Torr) reactivities to CO_2_ for the PCA and MCA with cerebral AI (C,D)**.

Cerebral AI for both the PCA and the MCA was highly correlated (*p* < 0.001) with systemic AI (Figure 5A). Furthermore, the curvilinear model fitted to the cerebral AI and age relationship was significant for both arteries (*p* < 0.001; Figure 5B). Figure 6 shows the relationships between responses in PCA *V*_mean_ to the visual stimulation and cerebral AI as well as the relationships between PCA and MCA hypercapnia reactivities and cerebral AI. The change in PCA *V*_mean_ (relative and absolute) in response to the visual stimulation was not related cerebral AI (*p* ≥ 0.128). Similarly, the correlations between absolute, and relative, PCA hypercapnia reactivity and cerebral AI were not significant (*p* ≥ 103). In contrast, there was a significant negative correlation between relative, and absolute, measures of MCA hypercapnia reactivities and cerebral AI (*p* ≤ 0.013) with lower reactivity at higher cerebral AIs.

## Discussion

In this study we assessed age-related changes in resting CBF through two cerebral arteries (PCA and MCA), as well as changes in CBF through the PCA and MCA in response to neuronal activation (via a visual stimulus) and hypercapnia in young and older healthy humans. The main findings were (1) resting PCA blood flow was not reduced with age, but resting MCA blood flow was lower in older subjects, (2) CBF responses to visual stimulation and hypercapnia were lower in older subjects; (3) cerebral and systemic AI increased with age and were highly correlated; and (4) cerebrovascular reactivity to hypercapnia through the MCA was inversely related to cerebral AI.

Resting CBF has been reported to decrease with advancing age (Kety, 1956; Leenders et al., 1990), but this is not a consistent finding (Yamaguchi et al., 1986). Reduced cerebral metabolism and brain atrophy are likely primary contributors to the age-related reduction in CBF (Leenders et al., 1990), but hemodynamic alterations such as increased arterial stiffness may also be involved (Zhu et al., 2011). In the current study, resting PCA blood flow was similar between the young and older groups, while the MCA blood flow was significantly lower in the older group. The lower resting MCA blood flow is consistent with prior studies utilizing TCD (Nishiyama et al., 1997; Ainslie et al., 2008; Secher et al., 2008; Galvin et al., 2010; Zhu et al., 2013), but the maintained resting PCA blood flow in the older group is in contrast to two previous studies that reported concurrent decreases in both PCA, and MCA, blood flow with aging (Muller and Schimrigk, 1994; Demirkaya et al., 2008). However, in the study by Muller and Schimrigk (1994) the age-related decline was more pronounced for the MCA than the PCA—29 vs. 17%. Oppositely, Demirkaya et al. (2008) reported a ~20% decrease in both MCA and PCA blood flow with increasing age. In conjunction with the findings by Muller and Schimrigk (1994), the observation of a maintained resting PCA blood flow, but a lower resting MCA blood flow, reflect potential regional differences in cerebrovascular changes with aging. These contrasting changes in PCA and MCA blood flow may be the result of the PCA being a smaller artery with, generally, lower blood flow compared to the MCA. As a result, the PCA likely has a reduced capacity to decrease flow (i.e., floor effect) in comparison to the MCA. An additional explanation of why aging may affect the PCA and MCA differently, is that the PCA and MCA bifurcate from different arteries (basilar artery vs. internal carotid artery, respectively) and perfuse differing volumes of brain tissue. The degree of atrophy with healthy aging varies across brain regions with the visual cortex being the most stable across the lifespan (Raz et al., 2005). However, the reduction in CBF with aging has been reported not to be related to brain atrophy (Chen et al., 2011). Interestingly, cerebral AI values were increased for both the MCA, and PCA, in the older group, but resting CBF was not related to AI for either artery (PCA: *R*^2^ = 0.08, *p* = 0.138; MCA: *R*^2^ = 0.04, *p* = 0.290; data not shown). Therefore, arterial stiffness does not appear to be involved in age-related changes in resting blood flow through these two arteries.

Although visual-evoked increases in blood flow were observed in both the PCA and MCA, the response observed in the PCA was much greater in both age groups. In young subjects, PCA blood flow increased ~14% while blood flow through the MCA increased only ~1%. Similarly, in older subjects, PCA blood flow increased ~8 vs. a ~3% increase in MCA blood flow. In contrast, hypercapnia caused similar relative increases in CBF through both the PCA and MCA (18% in the young group and ~14% in the older group). These contrary responses between the PCA and MCA to these two stimuli reflect the respective local and global responses evoked by the visual stimulation and hypercapnic tests (Tominaga et al., 1976; Aaslid, 1987).

The lower PCA blood flow response to the visual stimulation in the older group is similar to previous studies reporting aging-related declines in PCA blood flow responses to a visual stimulus (Niehaus et al., 2001; Zheng et al., 2003), but in contrast to other studies reporting no change with increasing age (Panczel et al., 1999; Rosengarten et al., 2003). The mechanism(s) underlying visually evoked blood flow responses is incompletely defined, but include a critical role of the neurovascular unit (i.e., astrocytes in combination with the neurons and blood vessels), as summarized in recent reviews (Iadecola, 2004; Drake and Iadecola, 2007; Iadecola and Nedergaard, 2007; Lok et al., 2007; Koehler et al., 2009). Thus, the observed decrease in PCA blood flow responses with aging may reflect a disruption of the neurovascular unit with aging resulting from neuronal loss and/or vascular remodeling (Panczel et al., 1999).

For cerebrovascular reactivity to hypercapnia, reports are also inconsistent regarding age-related alterations with studies reporting decreased (Nishiyama et al., 1997; Bakker et al., 2004), maintained (Kastrup et al., 1998; Ito et al., 2002; Galvin et al., 2010) and even enhanced hypercapnia reactivity with advancing age (Zhu et al., 2013). The lower PCA and MCA hypercapnic reactivity in the older group observed in the current study are in agreement with the prior studies reporting cerebrovascular reactivity to hypercapnia is decreased with advancing age.

A novel aspect of the current study is the observed decrease in both PCA and MCA reactivity to hypercapnia. Prior studies investigating changes in cerebrovascular reactivity to hypercapnia with aging via TCD have monitored only changes in MCA reactivity (Nishiyama et al., 1997; Kastrup et al., 1998; Ito et al., 2002; Bakker et al., 2004; Galvin et al., 2010; Zhu et al., 2013), thus ignoring potential regional differences that may occur. Therefore, the lower PCA and MCA hypercapnic reactivity in the older group signifies that, in contrast to the regional differences observed in age-related changes in resting CBF, the decreased cerebrovascular reactivity to hypercapnia is a global cerebral phenomenon. The mechanism responsible for the vascular responses to hypercapnia is thought to be the increase in H^+^ concentration in the cerebrospinal fluid which leads to relaxation of the smooth muscle around the cerebral vessels (Gotoh et al., 1961; Kontos et al., 1977a,b; Berne et al., 1981). As a result, the decline in hypercapnic reactivity in both the PCA and MCA reflect a homogenous decline in the capacity of the cerebrovasculature to dilate in response to hypercapnia.

As the mechanisms underlying the regulation of vascular responses to visual stimulation and hypercapnia are different, but both responses are decreased with age, there is a likely common factor that is altered with age contributing to the reduced evoked responses. The mechanical function of the vasculature is involved in both mechanisms and thus, is a likely candidate contributing to the two decreased evoked responses. Furthermore, Zhu et al. (2011) who applied a transfer function method to estimate cerebrovascular impedance, suggested arterial stiffness may contribute to attenuations in CBF with age. With aging, systemic arterial stiffening has been reported to increase in a curvilinear fashion with the greatest increase occurring between 30 and 60 years of age (Kelly et al., 1989; Mitchell et al., 2004; McEniery et al., 2005). Mitchell et al. (2004) reported a leveling off and even a slight decrease of the reflected pressure wave in individuals 50 years of age or older that was explained by an impedance matching due to marked increases in aortic stiffness compared to only slight increases in the periphery and, thus a reduction of wave reflection. PCA and MCA cerebral AIs were strongly correlated with systemic AI, and increased reflected waves were observed in both the velocity (i.e., cerebral), and pressure (i.e., systemic) waveforms within both arteries. This high degree of correlation between cerebral and system AI is similar to the studies by Kwater et al. (2009) and Xu et al. (2012) reporting significant correlations between MCA and systemic arterial stiffness indices. Based upon the observed high correlation between cerebral and systemic AI, a curvilinear function was fitted to the cerebral AI and age relationship. Although additional data is needed to fully support a curvilinear relationship between cerebral AI and age due to the gap of ~2 decades between our young and older groups, the curvilinear relationships plotted had moderate coefficients of determination (i.e., *R*^2^) for the PCA (0.45) and MCA (0.31). Moreover, consistent with the systemic arterial stiffness and age relationship (Kelly et al., 1989; Mitchell et al., 2004; McEniery et al., 2005) the greatest increase in cerebral AI occurred between 20 and 60 years of age. Hence, cerebral and system arterial stiffness may increase in parallel with healthy aging.

Although the negative relationship between the increase in PCA blood flow with visual stimulation and cerebral AI is in agreement with our hypothesis (i.e., increased arterial stiffness contributes to decreased blood flow responses), the relationship was not significant. Similarly, the negative relationship between the PCA hypercapnic reactivity and cerebral AI was not significant. In contrast, a lower MCA hypercapnic reactivity was related to an increased cerebral AI. These divergent findings for the hypercapnic reactivity between the PCA and MCA suggest MCA reactivity is more susceptible to arterial stiffening. The non-significant relationship between PCA blood flow responses to the visual and hypercapnic stimuli and cerebral AI may have resulted from the greater variability in the PCA response to hypercapnia and/or the PCA AI having a smaller distribution compared to the MCA AI. Another potential explanation is that AI magnitude is dependent upon wave reflections throughout the vascular bed resulting from vessel branching, changes in vessel wall diameter and/or material properties (Mitchell et al., 2011). Thus, changes in vascular morphology with aging will impact on the relationship between vascular reactivity and cerebral AI. With aging, there is a decrease in the number of downstream blood vessels from the PCA and MCA with a greater decline occurring within the MCA circulation (Bullitt et al., 2010). In contrast, there is a greater increase in vessel tortuosity within the MCA circulation compared to the PCA circulation with advancing age (Bullitt et al., 2010). As a result, the stronger relationship between vascular reactivity through the MCA and cerebral AI may be driven by an increase in vessel tortuosity that occurs with healthy aging.

Lastly, this study has some limitations, which need to be acknowledged. First, TCD measures blood velocity through the insonated artery and not absolute blood flow. However, the two are highly correlated (Brauer et al., 1998). Moreover, the diameter of the PCA during visual stimulation does not change (Aaslid, 1987), and the diameter of both, the PCA, and MCA, has been shown not to change significantly in response to moderate hypercapnia as employed in this study (Giller et al., 1993; Poulin and Robbins, 1996; Serrador et al., 2000; Willie et al., 2012). Thus, in this situation, changes in blood velocity will lead to a corresponding increase in absolute blood flow. As such, assessment of blood velocity through the PCA and MCA with TCD has been accepted as a reliable index of CBF. Secondly, the gold standard to assess vessel stiffness is pulse wave velocity measurement (Tomlinson, 2012). AI is straightforward to measure, by monitoring blood pressure or blood velocity waveforms; however wave reflections are not only dependent on arterial stiffness but also on the site of reflection. In addition, blood pressure is said to influence the vessel wall properties due to the distending pressure (Chirinos, 2012). However, subjects in the present study were assessed at rest and the chosen time periods used for AI calculations was assured to be as stable as possible.

In conclusion, CBF responses to both visual stimulation and hypercapnia decreased with advancing age with a concomitant increase in cerebrovascular stiffness. The decreases in PCA reactivity to a visual stimulus and hypercapnia were not related to increased cerebrovascular stiffness, whereas MCA hypercapnia reactivity decreased as cerebrovascular stiffness increased. Finally, analysis of the cerebral blood velocity waveform offers a non-invasive approach to assess vascular health and provide an index of arterial stiffness.

## Author contributions

Daniela Flück participated in study design, collected and analyzed TCD data from the older participants, drafted the manuscript, critically reviewed and revised the manuscript; Andrew E. Beaudin and Craig D. Steinback participated in study design, co-collected and co-analyzed TCD data from young participants, critically reviewed and revised the manuscript. Gopukumar Kumarpillai and Nandavar Shobha performed assessments on older participants, critically reviewed and revised the manuscript; Cheryl R. McCreary and Stefano Peca participated in study design, critically reviewed and revised the manuscript; Eric E. Smith conceived of the study, secured study funding, participated in study design, critically reviewed and revised the manuscript; and Marc J. Poulin conceived the study, secured study funding, supervised trainees (Daniela Flück, Andrew E. Beaudin, Craig D. Steinback) and acquisition and analysis of TCD data, critically reviewed and revised the manuscript for intellectual content. All authors approved the final version of the manuscript.

### Conflict of interest statement

The authors declare that the research was conducted in the absence of any commercial or financial relationships that could be construed as a potential conflict of interest.
